# Present State and Future Prospects of Pediatric Liver Transplantations

**DOI:** 10.31662/jmaj.2018-0024

**Published:** 2018-09-28

**Authors:** Hidetoshi Eguchi, Masaki Mori

**Affiliations:** 1 Department of Gastroenterological Surgery, Graduate School of Medicine, Osaka University, Osaka, Japan

**Keywords:** pediatric liver transplantation, deceased donor liver transplantation, living donor liver transplantation, hepatocyte transplantation

The world’s first liver transplantation was performed on a 3-year-old patient with biliary atresia by Starzl et al. in 1963 (Table 1)^(1)^. The postoperative outcome of liver transplantations that were initially performed using livers harvested from deceased donors remained poor at earlier periods despite various trials and errors. However, with the subsequent improvement of surgical procedures and the development of immunosuppressants, the postoperative outcome of liver transplantation has improved gradually. Currently, deceased donor liver transplantation (DDLT) is considered one of the standard treatments for various liver diseases. In 1988, Raia et al. performed the world’s first living donor liver transplantation (LDLT) ^(2)^. Although acceptable postoperative outcomes can be obtained by DDLT or LDLT, LDLT should be considered a last resort against the lack of organs from deceased donors because this method requires liver resection from healthy donors.

**Table 1.** Important Events of Liver Transplantation.

Year	Event
1963	World’s first liver transplantation
1988	World’s first living donor liver transplantation
1989	First living donor liver transplantation in Japan
1992	World’s first hepatocyte transplantation

Kasahara et al. published “Present state and future prospects of pediatric liver transplantations” in the JMA Journal ^(3)^. This article outlined the history of pediatric liver transplantations in Japan and revealed its current problems. Owing to severe organ shortage from deceased donors, LDLT has become the mainstream approach in Japan for both adult and pediatric liver transplantations. Donor death due to LDLT was reported in 2003 ^(4)^. The safety of donors is the most important issue in liver transplantation, particularly for adult LDLT, which requires a larger volume of liver graft than pediatric LDLT.

Among the 8347 liver transplants conducted in Japan as of 2017, there are only 447 DDLT cases, with the remainder of cases being dependent on LDLT. Therefore, compared with other countries, liver transplantation in Japan is characterized by a remarkably high proportion of LDLT operations. Among the total LDLT cases, pediatric LDLT cases account for 36.8% of the total number of cases, and this number is significantly higher than the proportion of LDLT cases (7.3%) in the United States. Kasahara et al. carefully concluded that this fact did not indicate that pediatric liver transplantation was actively performed in Japan but rather indicated that the transplantation had not been equally performed for adult patients who need liver transplantation ^(3)^.

Although Kasahara et al. insisted in the article that enlightenment was needed to increase DDLT, they also introduced two advantages of LDLT ^(3)^. The first advantage is that LDLT enables the transplantation of organs with better viability compared with deceased donor organs, which have been preserved in cold storage for a long time. The second advantage is that depending on the condition of the recipient, LDLT can be performed in an elective manner at the optimal time, even for patients whose condition suddenly deteriorated or patients who need urgent treatment. LDLT may be one of the important methods of liver transplantation, particularly in pediatric liver transplantation, even if organ shortage from deceased donors could be solved in the future.

Liver transplantation for children is characterized by small liver grafts. Therefore, designing small grafts is important to obtain a better postoperative outcome. The other typical point for pediatric liver transplantation is the limited number of deceased donor organs available from children, even in Europe and the United States where DDLTs are performed as a major organ transplantation procedure. Kasahara et al. performed active trials with hyper-reduced grafts for LDLT for neonates or split-liver transplantation ^(3)^. The National Center for Child Health and Development, where Kasahara is currently working, has been performing 60%–70% of the pediatric liver transplantations in Japan. They also perform hepatocyte transplantation in addition to their active trials in the surgical techniques described above. The world’s first hepatocyte transplantation was performed by Mito et al. in 1992 ^(5)^. In newborn metabolic diseases, the injection of frozen hepatocytes into the liver is a temporary but effective approach to compensate for enzyme deficiency. Hepatocyte transplantation is less invasive to patients, and it can be a promising method if sufficient sources of hepatocytes can be secured. As shown in the article, transplantable hepatocytes can be obtained from the surplus liver of living donors, and acquisition by means of regenerative medicine, including embryonic stem (ES) cells, induced pluripotent stem (iPS) cells, mesenchymal stem cells, or progenitor cells from human liver, is actively being investigated at present ^(6)^. Furthermore, studies are investigating organ regeneration in addition to hepatocyte regeneration. To overcome the organ shortage, new technologies need to be developed and advanced.

## Article Information

### Conflicts of Interest

None
